# Germline polymorphisms in the *Von Hippel-Lindau* and *Hypoxia-inducible factor 1-alpha* genes, gene-environment and gene-gene interactions and renal cell cancer

**DOI:** 10.1038/s41598-019-56980-0

**Published:** 2020-01-10

**Authors:** Jeroen A. A. van de Pol, Piet A. van den Brandt, Manon van Engeland, Roger W. L. Godschalk, Frederik-Jan van Schooten, Janneke G. F. Hogervorst, Leo J. Schouten

**Affiliations:** 1 Department of Epidemiology, GROW – School for Oncology and Developmental Biology, Maastricht University, Maastricht, the Netherlands; 2 Department of Epidemiology, Care and Public Health Research Institute (CAPHRI), Maastricht University, Maastricht, the Netherlands; 3 Department of Pathology, GROW – School for Oncology and Developmental Biology, Maastricht University Medical Centre, Maastricht, the Netherlands; 4 Department of Pharmacology & Toxicology, NUTRIM School of Nutrition and Translational Research in Metabolism, Maastricht University, Maastricht, the Netherlands; 5 Centre for Environmental Sciences, Hasselt University, Diepenbeek, Belgium

**Keywords:** Renal cell carcinoma, Cancer epidemiology, Cancer genetics

## Abstract

We investigated the relationship between germline single nucleotide polymorphisms (SNPs) in *Von Hippel-Lindau (VHL)* and *Hypoxia-inducible factor 1-alpha (HIF1A)*, and their gene-environment and gene-gene interactions, and clear-cell RCC (ccRCC) risk. Furthermore, we assessed the relationship between *VHL* SNPs and *VHL* promoter methylation. Three *VHL* polymorphisms and one *HIF1A* polymorphism were genotyped in the Netherlands Cohort Study. In 1986, 120,852 participants aged 55–69 completed a self-administered questionnaire on diet and lifestyle and toenail clippings were collected. Toenail DNA was genotyped using the Sequenom MassARRAY platform. After 20.3 years, 3004 subcohort members and 406 RCC cases, of which 263 ccRCC cases, were eligible for multivariate case-cohort analyses. V*HL*_rs779805 was associated with RCC (Hazard Ratio (HR) 1.53; 95% Confidence Interval (CI) 1.07–2.17) and ccRCC risk (HR 1.88; 95% CI 1.25–2.81). No associations were found for other SNPs. Potential gene-environment interactions were found between alcohol consumption and selected SNPs. However, none remained statistically significant after multiple comparison correction. No gene-gene interactions were observed between *VHL* and *HIF1A*. *VHL* promoter methylation was not associated with *VHL* SNPs. *VHL* SNPs may increase (cc)RCC susceptibility. No associations were found between gene-environment and gene-gene interactions and (cc)RCC risk and between *VHL* promoter methylation and *VHL* SNPs.

## Introduction

Genetic and epigenetic alterations in the *Von Hippel-Lindau* (VHL) gene are important drivers of carcinogenesis in clear-cell renal cell carcinoma (ccRCC)^1^. For sporadic ccRCC, biallelic inactivation of *VHL* because of rare, but highly penetrant, somatic mutations is relatively common^2,3^. Previous studies have estimated that 50–82% of patients with sporadic ccRCC have a mutation in the *VHL* gene^4–8^. The *VHL* gene encodes the VHL tumor suppressor protein (pVHL). Inactivation of pVHL leads to the unchecked accumulation of hypoxia-inducible factor 1 alpha (HIF1A), which facilitates oxygen delivery, adaptation to oxygen deprivation and angiogenesis^1,9^. Therefore, genetic or epigenetic alterations in *VHL* and *HIF1A* may lead to enhanced cell survival and carcinogenesis.

In contrast to the rare, but highly penetrant, sequence alterations leading to *VHL* loss, some germline Single Nucleotide Polymorphisms (SNPs) are highly frequent, but have a low penetrance. In general, SNPs account for many different phenotypes as they may alter disease susceptibility by affecting the gene’s function^10^. Genome-wide association studies (GWAS) have not found an association with *VHL* and *HIF1A* loci^11–18^. However, candidate gene studies have found conflicting evidence on the relationship between *VHL* SNPs and (cc)RCC risk, with some studies indicating a positive association^19,20^, while others indicate no association^21^. In previous studies, *HIF1A* SNPs have been associated with RCC prognosis, but not with (cc)RCC development^21,22^.

Previous studies have indicated the importance of assessing the interplay between genetic, epigenetic and environmental triggers when assessing ccRCC risk. Moore *et al*. found increased promoter hypermethylation in sporadic ccRCC when certain *VHL* polymorphisms were present^23^. In addition, multiple studies have indicated potential gene-environment interactions between germline SNPs and environmental factors in RCC^24–28^. To our knowledge, the relationship between established environmental risk factors associated with RCC risk, namely smoking, hypertension, obesity and alcohol consumption^29^, and *VHL* and *HIF1A* SNPs remains unstudied.

Therefore, we investigated the relationship between three selected germline *VHL* SNPs and one *HIF1A* SNP and (cc)RCC risk in the Netherlands Cohort Study on diet and cancer (NLCS). In addition, interactions between *VHL* and *HIF1A* SNPs and smoking, hypertension, body mass index (BMI) and alcohol consumption were studied. Lastly, we investigated the association between *VHL* promoter methylation and *VHL* SNPs.

## Results

After excluding participants with missing values for predefined confounders 3004 subcohort members and 406 RCC cases, of which 263 ccRCC cases, were included in the analyses. The proportion of men was higher in both RCC and ccRCC cases when compared to the subcohort (Table 1). In addition, cases were more often smokers and were more often diagnosed with hypertension when compared to the subcohort.

**Table 1:** Baseline characteristics of the subcohort and renal cell carcinoma (RCC) and clear cell renal cell carcinoma (ccRCC) cases; Netherlands Cohort Study on diet and cancer, 1986–2006.

Baseline characteristics (mean (SD))	Subcohort members	RCC	ccRCC
Total (n)	3004	406	263
Age (y)	61.3 (4.2)	60.7 (3.9)	60.6 (3.9)
Male sex (%)	49.6	65.8	64.3
Tumor stage (%)^a^			
Stage 1/2	—	49.8	51.0
Stage 3/4	—	38.2	39.5
Undefined	—	12.1	9.5
Cigarette smoking status (%)			
Never smoker	36.8	26.9	26.6
Former smoker	36.4	43.8	45.3
Current smoker	26.9	29.3	28.1
Smoking intensity (cig/d)^b^	15.2 (10.2)	17.0 (11.7)	16.2 (10.5)
Smoking duration (y)^b^	31.5 (12.2)	32.0 (11.6)	31.4 (11.3)
Hypertension (%)	26.4	33.5	33.5
BMI (kg/m^2^)	25.0 (3.1)	25.4 (3.0)	25.5 (2.9)
Alcohol consumption (g ethanol/d)^c^	13.7 (15.0)	15.4 (15.3)	14.9 (14.7)
Energy intake (kcal)	1915 (505)	1998 (529)	1994 (517)
Diuretic medication (%)	11.0	14.0	15.2
Antihypertensive medication (%)	20.5	24.6	26.2

The subcohort includes 15 RCC cases, of which 11 ccRCC cases. Solely participants with complete information for main exposures are included in this table. SD = Standard deviation, RCC = renal cell carcinoma, ccRCC = clear cell renal cell carcinoma, BMI = Body Mass Index.

^a^According to the TNM version used at time of diagnosis by the Netherlands Cancer registry.

^b^In former and current smokers only.

^c^In alcohol consumers only.

Genotype and allele frequencies for the four selected SNPs in subcohort members of the NLCS are presented in Supplementary Table 1. All selected SNPs adhered to the Hardy-Weinberg Equilibrium. Only *VHL_*rs779805 had a minor allele frequency (MAF) above 25% and is, therefore, assessed primarily using additive models.

### Main SNP effects

In both age- and sex-adjusted analyses and multivariable-adjusted analyses, an association with (cc)RCC risk was observed for SNPs in *VHL_*rs779805, but not for SNPs in *VHL_*rs1642739, *VHL_*rs265318 and *HIF1A_*rs2301111 (Table 2). In multivariable-adjusted analyses individuals carrying the AG (vs. AA) genotype of *VHL_*rs779805 had a statistically significantly increased RCC risk (HR 1.32, 95%CI 1.06–1.66), and the GG (vs. AA) genotype was associated with a statistically significantly increased RCC risk (HR 1.53, 95%CI 1.07–2.17). In addition, a statistically significant per-allele *p* for trend was observed (p = 0.004). In multivariable-analyses for ccRCC risk, the AG (vs. AA) genotype for *VHL_*rs779805 was associated with a statistically significantly increased ccRCC risk (HR 1.35, 95% CI 1.02–1.78), as was the GG (vs. AA) genotype of *VHL_*rs779805 (HR 1.88, 95%CI 1.25–2.81).

**Table 2:** Association between *VHL* and *HIF1A* Single Nucleotide Polymorphisms (SNPs) and (clear cell) renal cell carcinoma status; Netherlands Cohort Study on diet and cancer, 1986–2006.

*SNP*	HWE^a^	Subcohort person-years	Renal cell carcinoma				Clear-cell renal cell carcinoma			
			No. cases	HR^b, c^ (CI 95%)	HR_adjusted_^c, d^ (CI 95%)	*p* for trend^e^	No. cases	HR^b, c^ (CI 95%)	HR_adjusted_^c, d^ (CI 95%)	*p* for trend^e^
*VHL_*rs1642739^f^	0.291									
GG		39067	299	1 (reference)	1 (reference)		190	1 (reference)	1 (reference)	
GT		11262	103	1.19 (0.93–1.52)	1.18 (0.92–1.51)		71	1.29 (0.97–1.72)	1.29 (0.96–1.73)	
TT		993	4	0.50 (0.18–1.40)	0.50 (0.18–1.41)		2	0.39 (0.10–1.64)	0.40 (0.10–1.66)	
T				1.06 (0.86–1.29)	1.05 (0.86–1.29)	0.635		1.11 (0.87–1.41)	1.11 (0.87–1.41)	0.410
GT + TT		12255	107	1.13 (0.89–1.44)	1.13 (0.89–1.43)		73	1.22 (0.91–1.62)	1.21 (0.91–1.62)	
*VHL_*rs779805	0.598									
AA		23908	157	1 (reference)	1 (reference)		98	1 (reference)	1 (reference)	
AG		22432	198	1.33 (1.06–1.66)	1.32 (1.06–1.66)		126	1.36 (1.03–1.79)	1.35 (1.02–1.78)	
GG		4982	51	1.50 (1.06–2.11)	1.53 (1.07–2.17)		39	1.83 (1.23–2.73)	1.88 (1.25–2.81)	
G				1.25 (1.07–1.46)	1.26 (1.08–1.47)	0.004		1.35 (1.12–1.63)	1.36 (1.13–1.65)	0.001
AG + GG		27414	249	1.36 (1.10–1.69)	1.36 (1.10–1.69)		165	1.45 (1.11–1.88)	1.44 (1.11–1.88)	
*VHL_*rs265318^f^	0.965									
AA		41066	321	1 (reference)	1 (reference)		206	1 (reference)	1 (reference)	
AC		9686	82	1.10 (0.85–1.43)	1.09 (0.83–1.42)		54	1.13 (0.82–1.55)	1.12 (0.81–1.54)	
CC		570	3	0.62 (0.19–2.07)	0.65 (0.19–2.20)		3	0.97 (0.29–3.23)	1.03 (0.30–3.54)	
C				1.03 (0.82–1.30)	1.03 (0.81–1.30)	0.809		1.09 (0.83–1.45)	1.09 (0.82–1.45)	0.534
AC + CC		10256	85	1.07 (0.83–1.38)	1.06 (0.82–1.38)		57	1.12 (0.82–1.52)	1.11 (0.81–1.52)	
*HIF1A_*rs2301111^f^	0.581									
CC		32553	257	1 (reference)	1 (reference)		170	1 (reference)	1 (reference)	
CG		16741	138	1.06 (0.85–1.33)	1.06 (0.84–1.32)		88	1.02 (0.78–1.34)	1.02 (0.78–1.34)	
GG		2028	11	0.76 (0.40–1.44)	0.77 (0.41–1.47)		5	0.52 (0.21–1.29)	0.52 (0.21–1.30)	
G				0.99 (0.83–1.19)	0.99 (0.83–1.20)	0.956		0.93 (0.74–1.16)	0.92 (0.74–1.16)	0.486
CG + GG		18769	149	1.03 (0.83–1.28)	1.03 (0.83–1.28)		93	0.97 (0.75–1.27)	0.97 (0.74–1.27)	

Reference/minor alleles for the selected SNPs in the NLCS: VHL_rs1642739 – G/T; VHL_rs779805 – A/G; VHL_rs265318 – A/C; HIF1A_rs2301111 – C/G.

^a^Hardy-Weinberg Equilibrium as tested with the Pearson χ^2^-test based on the distribution of genotypes in the subcohort as presented in Supplementary Table 1.

^b^Models adjusted for age (y, continuous) and sex (man/woman).

^c^Models include a time-varying covariable for age due to a violation of the proportional hazards assumption.

^d^Models adjusted for age (y, continuous), sex (man/woman), hypertension (yes/no), smoking status (never, former, current), smoking duration (y, centered), smoking intensity (cig/d, centered), BMI (kg/m^2^, continuous) and alcohol consumption (g ethanol/d, continuous).

^e^The per-allele *p* for trend is based on the multivariable-adjusted model.

^f^Minor allele frequency < 0.25.

### Gene-environment interactions

In multivariable-adjusted models for RCC risk, potential gene-environment interactions were observed between *VHL_*rs1642739*, VHL_*rs779805 and *HIF1A_*rs2301111 SNPs and alcohol consumption (Table 3). A weak inverse association between alcohol consumption (per 5 g/day) and RCC risk was observed in participants carrying the rare genotype for *VHL_*rs1642739 and *VHL_*rs779805, but not in participants carrying the wild-type genotype. For carriers of the wild-type *HIF1A*_rs2301111 genotype a weak inverse association was observed between alcohol consumption and RCC risk, but not for individuals carrying the rare genotype. No interaction was observed between either of the selected SNPs and self-reported hypertension (yes, no), smoking status (never, former, current) and BMI (per kg/m^2^) for RCC risk. For ccRCC, a potential interaction between *VHL_*rs779805 SNPs and alcohol consumption was observed. However, after correction for multiple comparisons using the adaptive Benjamini-Hochberg method none of the potential gene-environment interactions maintained statistical significance^30^.

**Table 3:** Multivariable-adjusted gene-environment interactions for renal cell carcinoma (RCC) and clear-cell renal cell carcinoma; Netherlands Cohort Study on diet and cancer, 1986–2006.

Gene		Wild genotype			Rare genotype			*P* for interaction^c^	FDR *p*-threshold
Environment		Subcohort person-years	No. cases	HR_adjusted_^a,b^ (CI95)	Subcohort person-years	No. cases	HR_adjusted_^a,b^ (CI95)		
**Renal cell carcinoma**									
*VHL_*rs1642739		*GG*			*GT* + *TT*				
Hypertension	No	28949	198	1 (reference)	9088	72	1 (reference)		
	Yes	10118	101	1.48 (1.13–1.92)	3167	35	1.48 (0.95–2.32)	0.968	0.100
Smoking status^d^	Never	15367	72	1 (reference)	4667	37	1 (reference)		
	Former	13829	135	1.41 (0.97–2.04)	4558	43	1.26 (0.74–2.14)		
	Current	9871	92	1.41 (0.97–2.05)	3029	27	1.13 (0.64–1.99)	0.136	0.031
BMI	Per kg/m^2^	39067	299	1.05 (1.01–1.10)	12255	107	1.00 (0.94–1.06)	0.212	0.044
Alcohol consumption	Per 5 g/day	39067	299	1.00 (0.96–1.05)	12255	107	0.91 (0.82–1.02)	0.023	0.006
*VHL_*rs779805		*AA*			*AG + GG*				
Hypertension	No	17764	109	1 (reference)	20273	161	1 (reference)		
	Yes	6145	48	1.34 (0.92–1.93)	7140	88	1.55 (1.16–2.08)	0.433	0.063
Smoking status^d^	Never	9542	37	1 (reference)	10492	72	1 (reference)		
	Former	8471	72	1.31 (0.80–2.13)	9916	106	1.37 (0.93–2.02)		
	Current	5895	48	1.38 (0.82–2.31)	7005	71	1.29 (0.87–1.91)	0.330	0.050
BMI	Per kg/m^2^	23908	157	1.04 (0.98–1.11)	27414	249	1.04 (0.99–1.08)	0.833	0.081
Alcohol consumption	Per 5 g/day	23908	157	1.02 (0.97–1.06)	27414	249	0.95 (0.90–1.01)	0.024	0.013
*VHL_*rs265318		*AA*			*AC + CC*				
Hypertension	No	30540	211	1 (reference)	7497	59	1 (reference)		
	Yes	10526	110	1.52 (1.18–1.96)	2759	26	1.32 (0.79–2.20)	0.553	0.069
Smoking status^d^	Never	16103	85	1 (reference)	3932	24	1 (reference)		
	Former	14813	141	1.32 (0.94–1.86)	3574	37	1.38 (0.74–2.60)		
	Current	10150	95	1.28 (0.90–1.83)	2750	24	1.43 (0.76–2.71)	0.909	0.088
BMI	Per kg/m^2^	41066	321	1.05 (1.01–1.09)	10256	85	0.99 (0.92–1.07)	0.131	0.025
Alcohol consumption	Per 5 g/day	41066	321	0.99 (0.95–1.03)	10256	85	0.95 (0.85–1.06)	0.359	0.056
*HIF1A*_rs2301111		*CC*			*CG + GG*				
Hypertension	No	24090	170	1 (reference)	13947	100	1 (reference)		
	Yes	8462	87	1.48 (1.12–1.97)	4822	49	1.53 (1.04–2.24)	0.934	0.094
Smoking status^d^	Never	12704	77	1 (reference)	7330	32	1 (reference)		
	Former	11620	108	1.17 (0.80–1.70)	6767	70	1.77 (1.07–2.92)		
	Current	8229	72	1.05 (0.71–1.54)	4672	47	2.01 (1.19–3.40)	0.167	0.038
BMI	Per kg/m^2^	32553	257	1.04 (0.99–1.08)	18769	149	1.05 (0.99–1.11)	0.823	0.075
Alcohol consumption	Per 5 g/day	32553	257	0.96 (0.91–1.00)	18769	149	1.04 (0.97–1.11)	0.024	0.019
**Clear-cell renal cell carcinoma**									
*VHL_*rs1642739		*GG*			*GT + TT*				
Hypertension	No	28949	124	1 (reference)	9088	51	1 (reference)		
	Yes	10118	66	1.51 (1.09–1.09)	3167	22	1.38 (0.80–2.38)	0.614	0.069
Smoking status^d^	Never	15367	47	1 (reference)	4667	23	1 (reference)		
	Former	13829	90	1.56 (1.01–2.41)	4558	29	1.36 (0.73–2.56)		
	Current	9871	53	1.33 (0.83–2.12)	3029	21	1.34 (0.69–2.59)	0.398	0.050
BMI	Per kg/m^2^	39067	190	1.06 (1.01–1.11)	12255	73	1.00 (0.93–1.07)	0.103	0.019
Alcohol intake	Per 5 g/day	39067	190	0.99 (0.95–1.05)	12255	73	0.90 (0.77–1.04)	0.085	0.012
*VHL_*rs779805		*AA*			*AG + GG*				
Hypertension	No	17764	66	1 (reference)	20273	109	1 (reference)		
	Yes	6145	32	1.45 (0.92–2.28)	7140	56	1.47 (1.03–2.09)	0.929	0.094
Smoking status^d^	Never	9542	21	1 (reference)	10492	49	1 (reference)		
	Former	8471	49	1.65 (0.89–3.06)	9916	70	1.42 (0.91–2.22)		
	Current	5895	28	1.64 (0.84–3.21)	7005	46	1.19 (0.74–1.91)	0.200	0.038
BMI	Per kg/m^2^	23908	98	1.06 (0.98–1.13)	27414	165	1.04 (0.99–1.09)	0.853	0.088
Alcohol intake	Per 5 g/day	23908	98	1.02 (0.96–1.08)	27414	165	0.94 (0.87–1.01)	0.020	0.006
*VHL_*rs265318		*AA*			*AC + CC*				
Hypertension	No	30540	135	1 (reference)	7497	40	1 (reference)		
	Yes	10526	71	1.53 (1.12–2.08)	2759	17	1.25 (0.68–2.31)	0.544	0.056
Smoking status^d^	Never	16103	54	1 (reference)	3932	16	1 (reference)		
	Former	14813	95	1.47 (0.98–2.22)	3574	24	1.46 (0.70–303)		
	Current	10150	57	1.24 (0.80–1.94)	2750	17	1.59 (0.76–3.33)	0.962	0.100
BMI	Per kg/m^2^	41066	206	1.05 (1.00–1.10)	10256	57	1.01 (0.94–1.09)	0.191	0.031
Alcohol consumption	Per 5 g/day	41066	206	0.99 (0.94–1.04)	10256	57	0.90 (0.76–1.06)	0.161	0.025
*HIF1A*_rs2301111		*CC*			*CG + GG*				
Hypertension	No	24090	114	1 (reference)	13947	61	1 (reference)		
	Yes	8462	56	1.43 (1.02–2.01)	4822	32	1.61 (1.01–2.57)	0.640	0.075
Smoking status^d^	Never	12704	48	1 (reference)	7330	22	1 (reference)		
	Former	11620	74	1.34 (0.86–2.08)	6767	45	1.80 (0.98–3.32)		
	Current	8229	48	1.18 (0.74–1.88)	4672	26	1.68 (0.86–3.27)	0.657	0.081
BMI	Per kg/m^2^	32553	170	1.03 (0.98–1.08)	18769	93	1.06 (0.99–1.13)	0.545	0.063
Alcohol consumption	Per 5 g/day	32553	170	0.96 (0.90–1.02)	18769	93	1.01 (0.93–1.10)	0.319	0.044

^a^Models include a time-varying covariable for age due to a probable violation of the proportional hazards assumption.

^b^Models adjusted for age (y, continuous), sex (man/woman), hypertension (yes/no), smoking status (never, former, current), smoking duration (y, centered), smoking intensity (cig/d, centered), BMI (kg/m^2^, continuous) and alcohol consumption (g ethanol/d, continuous) when applicable.

^c^No interaction was significant after false discovery rate correction.

^d^Additionally adjusted for smoking duration (centered, years) and smoking intensity (centered, cig/d).

In sensitivity analyses, a potential gene-environment interaction was apparent between categorized alcohol consumption (0 g/d, 0.1–4 g/d, 5–14 g/d, 15–29 g/d and 30+ g/d) and *VHL_*rs164273 status for ccRCC risk (p = 0.009; Supplementary Table 2). The direction of associations for *VHL*_rs779805 was similar to main analyses using alcohol consumption (per 5 g/day). Sensitivity analyses between smoking status (ever/never), hypertension (no self-reported hypertension or no self-reported antihypertensive medication, hypertension with self-reported hypertensive medication) and BMI (<20 kg/m^2^, 20–<25 kg/m^2^, 25–<30 kg/m^2^ and 30+ kg/m^2^) and SNP status showed similar associations compared to main gene-environment analyses (Supplementary Table 2). Similar to main analyses, no sensitivity analysis remained statistically significant after multiple comparison correction.

### Gene-gene interactions

No gene-gene interactions, as tested with the Wald χ^2^_-_test, were found between the three selected *VHL* SNPs and *HIF1A_*rs2301111 for both RCC (p = 0.310, p = 0.321 and p = 0.514 for *VHL_*rs1642739*, VHL_*rs779805 and *VHL_*rs265318, respectively) and ccRCC (p = 0.762, p = 0.442 and p = 0.978 for *VHL_*rs1642739*, VHL_*rs779805 and *VHL_*rs265318, respectively).

### Association between SNPs and *VHL* promoter methylation status

In total, information on VHL promoter methylation was available from 253 ccRCC cases. Among ccRCC cases, 19 (7.5%) participants had a methylated CpG island in the *VHL* promoter region of which 13 had at least one mutant allele for the selected *VHL* SNPs (Supplementary Table 3). *VHL* promoter methylation was apparent in three, twelve and two participants for the rare genotype of *VHL_*rs1642739 (GG vs. GT + TT), *VHL*_rs779805 (AA vs. AG + GG) and *VHL*_rs264318 (AA vs. AC + CC), respectively. In multivariable-adjusted analyses a non-significant inverse association was observed between both *VHL*_rs1642739 (HR 0.45, 95%CI 0.12–1.69) and *VHL*_rs265318 (HR 0.38, 95%CI 0.07–2.00) and *VHL* promoter methylation in ccRCC cases. No association was observed for the *VHL_*rs779805 SNP (HR 0.99, 95%CI 0.37–2.69).

## Discussion

In this study, a statistically significantly increased RCC risk was found for individuals that carry genotypes with at least one variant allele for the *VHL_*rs779805 SNP. This association was especially pronounced for ccRCC risk. No association was found for *VHL_*rs164239*, VHL_*rs265318 and *HIF1A_*rs2301111. After adjustment for multiple comparisons, no statistically significant gene-environment interactions were found between the selected SNPs and smoking, hypertension, BMI and alcohol for both RCC and ccRCC cases. No gene-gene interactions were found between selected *VHL* SNPs and the *HIF1A* SNP.

Several studies have assessed the relationship between the *VHL_*rs779805 SNP and sporadic RCC^19–21^. Lv *et al*. found an association between the germline SNP *VHL_*rs779805 and RCC risk. Similarly, we found a statistically significant positive trend for the G allele and a positive association between the GG genotype for *VHL_*rs779805 and RCC risk^20^. The aforementioned studies did not report associations between *VHL* SNPs and ccRCC risk. In our study, rare *VHL_*rs779805 genotypes had a stronger association with ccRCC risk than with RCC risk. This might indicate that *VHL* polymorphisms lead to an increased susceptibility for ccRCC in particular. To our knowledge, no other study has investigated the relationship between *VHL_*rs1642739, *VHL_*rs265318 and *HIF1A_*rs2301111 and (cc)RCC risk. In this study, no association was found between (cc)RCC risk and *VHL_*rs1642739, *VHL_*rs265318 or *HIF1A_*rs2301111.

Multiple studies have assessed gene-environment interactions in RCC and ccRCC. RCC risk has been found to be associated with interactions between alcohol consumption and *ADH7*^26^; sodium and hypertension and *AGTR*, *AGT* and *ACE*^31^; calcium and vitamin D intake and *RXRA*^28^; tobacco smoking and *NAT2*, *CYP1A1* and *GSTM1*^25^; and meat-cooking mutagens and *ITPR2* and *EPAS1*^27^. To our knowledge, we are the first to study gene-environment interactions between the selected *VHL* and *HIF1A* SNPs and smoking, hypertension, BMI and alcohol consumption. Solely the interaction between *VHL_*rs779805 and alcohol consumption was associated with both RCC and ccRCC risk. However, this association did not maintain statistical significance after correction for multiple comparisons with the adaptive Benjamini-Hochberg method. Dominant models were used for all gene-environment analyses because of the low MAF of most included SNPs. However, SNPs may not have adhered to a dominant model, as there may be differences in disease susceptibility between heterozygous and homozygous rare genotypes, as was found for *VHL_*rs779805 (Table 2). This exemplifies that our gene-environment analyses may have been hampered by the inability to assess interactions per genotype. Further research is needed to ascertain the interaction between alcohol and *VHL* SNP status on (cc)RCC risk.

Disruptions in the *VHL* tumor suppressor gene are thought to play a role in the constitutive activation of hypoxia-inducible factors, as regulated in part by *HIF1A*, which may lead to carcinogenesis^1^. Therefore, it is plausible for gene-gene interactions to occur. However, in this study, we did not find gene-gene interactions between selected *VHL* and *HIF1A* SNPs on the risk of developing (cc)RCC.

Previous studies have found a relationship between VHL promoter hypermethylation and SNPs in *VHL_*rs779805 in sporadic ccRCC cases^6,23^. Moore *et al*. also reported a positive association between promoter hypermethylation and *VHL_*rs265318 and *VHL_*rs1642739. In contrast, we found no association between promoter methylation status and *VHL_*rs779805 in ccRCC cases. *VHL_*rs1642739 and *VHL*_rs265318 seemed inversely associated with *VHL* promoter methylation in ccRCC cases. However, this association was based on a limited sample size. While the number of cases with known promoter methylation status was similar in size to the study of Moore *et al*., our study had a smaller proportion of cases with *VHL* promoter methylation (7.5% vs. 9.8%)^23^. Banks *et al*. reported an even higher proportion of sporadic ccRCC cases with a methylated *VHL* promoter (20.4%), but had a smaller study population^6^. In general, there are large differences in the proportion of methylated *VHL* promoters per SNP between studies, which may explain these unstable point estimates^23^. Therefore, more research with a larger number of sporadic ccRCC cases is needed to elucidate the relationship between *VHL* promoter methylation and *VHL* SNPs.

At present, genome-wide association studies (GWAS) have identified multiple novel risk loci that may contribute to RCC susceptibility. Interestingly, SNPs in the *VHL* and *HIF1A* genes have not (yet) been identified as potential risk variants, while there is a biological plausibility for the involvement of these genes based on current evidence on the development of RCC^2,9^. For example, risk loci have been identified in *EPAS1*^11,13,17,18^, which is known to be involved in the VHL-HIF-1 pathway^32^. While we found no evidence for an association between three of our selected SNPs, *VHL*_rs779805 was associated with an increased risk of RCC. This finding was in line with two prior published studies, in which a potential association between *VHL_*rs779805 and RCC risk was found^19,20^. While this particular SNP is present on commonly used SNP arrays, this SNP remains unidentified in large-scale GWAS studies^11–18^. It is estimated that the currently available risk loci for RCC account for approximately 10% of the familial risk for RCC^11^. Therefore, it may well be possible for minor susceptibility loci to remain unidentified in GWAS studies, due to their tendency to convey small-to-moderate changes in risk, while major susceptibility loci are detectable in the stringent false discovery rate correction criteria of GWAS studies. This could be a reason why SNPs like *VHL*_rs779805 may remain unidentified, unless alternative methodologies are employed^11^. As a result, there is ample opportunity to discover new, rarer, RCC risk variants in future research. Additional evidence on risk loci from GWAS studies, combined with extensive information on direct effects, environmental factors and other potential modulators of disease etiology from candidate SNP studies, should lead to new insights into the biology of RCC to further the potential for new prevention, early detection and intervention strategies to be employed^11^.

This study also has several strengths. Strengths of this study were the detailed questionnaire information, the long duration and the histological revision of RCC cases by two experienced pathologists. Furthermore, cases in our study were obtained prospectively from a population of 120,852 men and women from 204 Dutch municipalities. Combined with the completeness of follow-up, we assume that these cases are a representative of kidney cancer cases in the Netherlands at the time.

In conclusion, this study confirmed the association between germline SNP *VHL_*rs779805 with RCC risk. In addition, a slightly stronger association for ccRCC was found compared to RCC. Potential gene-environment interactions were found between alcohol and *VHL* SNPs. However, results did not remain statistically significant after correction for multiple comparisons. No gene-gene interactions were observed between the *VHL* and *HIF1A* SNPs. Lastly, tumor promoter methylation was not significantly associated with *VHL* SNPs.

## Methods

### Study design

The NLCS is a nation-wide prospective cohort study initiated in September 1986 with the inclusion of 120,852 participants aged 55–69 years to study the relationship between diet and cancer. The study design has been described in detail elsewhere^33^. In short, a case-cohort design was used for efficiency in data processing and follow-up for vital status. Cases were derived from the entire cohort, whereas a subcohort of 5000 participants, consisting of 2411 men and 2589 women, was randomly sampled at baseline to estimate person years at risk for the entire cohort. The subcohort was followed up biennially for migration and vital status information by contacting participants and using computerized municipalities registries. Using the subcohort, person-years at risk were calculated from baseline until registration of RCC, or until date of censoring by death, emigration, loss to follow-up or end of follow-up, whichever occurred first. Cancer follow-up for the full cohort was conducted by computerized record linkage with the Netherlands Cancer Registry (NCR), the Netherlands Pathology Registry (PALGA), and causes of death registry maintained by Statistics Netherlands (CBS)^34^. Follow-up for vital status of the subcohort was nearly 100% complete after 20.3 years. The completeness of cancer follow-up is estimated to be over 96%^35^.

Individuals with prevalent cancer, excluding skin cancer, at baseline were excluded. After 20.3 years of follow-up, 608 RCC cases were identified (International Classification of Diseases for Oncology 3 (ICD-O-3):C64). Histologically confirmed epithelial RCC cases were eligible for the collection of formalin-fixed paraffin-embedded (FFPE) tumor tissue. Tumor blocks were collected for 454 out of 568 eligible cases (80%). Two experienced pathologists revised the tumor histology according to the WHO-classification of RCC tumors^36^. Based on this revision 366 (81%) of the cases with available tumor blocks were classified as ccRCC cases, 60 (13%) as papillary RCC cases, 15 (3.3%) chromophobe RCC cases, and 13 (2.9%) other or undefined RCC cases.

### Ethics statement

Individuals invited to participate in the NLCS received an invitation letter with details on the study and the use of their data. In addition, they received the baseline questionnaire, which included an envelope for returning toenail clippings. By completing and returning the baseline questionnaire, individuals consented to participate in the NLCS (response rate 35.5%). Individuals were informed about the possibility to end their participation at any time, at which point all their data would be removed. All methods were performed in accordance with the relevant guidelines and regulations that were applicable at that time (1986). The institutional review boards of Maastricht University (Maastricht) and the Netherlands Organization for Applied Scientific Research TNO (Zeist) approved the NLCS (February 2, 1985 and January 6, 1986, respectively). The institutional review board of Maastricht University (Maastricht) later re-evaluated the original approval of the study protocol and procedures (2010). Based on the re-evaluation the institutional review board amended the original approval to include the genotyping of SNPs (April 12, 2010). Participants did not provide written informed consent to the sharing of data.

### Gene and SNP selection

Genes and SNPs related to RCC risk were selected through literature search. Priority was given to SNPs with a MAF ≥ 20% in Caucasians and primers had to be compatible with RAAS-pathway SNPs present on the multiplex assay^31^. Consequently, three *VHL* SNPs (rs779805, rs265318 and rs1642739) and one *HIF1A* SNP (rs2301111) were selected. All included *VHL* SNPs were selected based on their association with *VHL* promoter methylation in previous research^23^. The included *HIF1A* tag-SNP had the highest MAF of the *HIF1A* SNPs compatible with the assay.

### Tissue collection and DNA isolation

Approximately 90,000 participants provided toenail clippings at baseline, which have been shown to be a valid source of DNA for the genotyping of germline genetic variants^37^. DNA was isolated according to the DNA isolation protocol by Cline *et al*.^38^. To increase the number of cases with available DNA, DNA was isolated from FFPE healthy tissue, as described by van Houwelingen *et al*.^5^, for 67 RCC cases without toenail clippings. There were no substantial quality differences between DNA samples from toenail and FFPE healthy tissue^31^. In total, 3582 (75%) subcohort members and 502 (83%) RCC cases were genotyped.

SNP genotyping was performed on the Sequenom MassARRAY platform using the iPLEX assay (Sequenom Inc., Hamburg, Germany), as described previously^31^. This method provides suitable SNP call rates and reproducibility using toenail DNA^37^.

DNA methylation of the CpG island of the *VHL* gene promoter region, of which methylation has been associated with inhibition of *VHL* gene expression^39^, in RCC tumor blocks was determined by chemical modification of genomic DNA with sodium bisulfite and subsequent methylation-specific PCR analysis (MSP) as previously described elsewhere^40–42^. MSP primer design was based on the MBD-affinity massive parallel sequencing data. Detailed information on primer sequences and MSP conditions are available elsewhere^24^.

### Questionnaire information

All participants completed a mailed, self-administered, questionnaire on diet and other cancer risk factors for cancer at baseline (1986)^43^. Information on dietary habits was obtained through a 150-item, semi-quantitative food frequency questionnaire (FFQ) focusing on habitual consumption of food and beverages during the year preceding baseline.

Cigarette smoking status, frequency and duration were based on self-reported information. Participants reported hypertension as diagnosed by a physician, preceding baseline. Participants were asked to report the use of any drugs that they used longer than 6 months. From this information, the use of antihypertensive medication was extracted. BMI was calculated using self-reported height and weight from the baseline questionnaire. Questions on beer, red wine, white wine, sherry, fortified wines, liqueur, and liquor were used to assess the consumption of alcohol. Participants who consumed alcoholic beverages less than once a month were considered non-users. Standard glass sizes were defined as 200 ml for beer, 105 ml for wine, 80 ml for sherry, and 45 ml for both liqueur and liquor^44^. These values corresponded to 8, 10, 11, 7 and 13 grams of alcohol, respectively. Mean daily alcohol consumption was calculated by multiplying the consumption frequency and the standardized item unit.

### Statistical analyses

Cox proportional hazards models were used to estimate age- and sex-adjusted and multivariable-adjusted hazard ratios (HR) and 95% confidence intervals (CIs). *A priori* selected covariables in the multivariable-adjusted model were BMI (kg/m^2^, continuous), hypertension (yes,no), cigarette smoking status (never, former, current), intensity (cig/d, centered; continuous), duration (years, centered; continuous) and alcohol consumption (g/d, continuous).

The most common allele was used as the reference allele. Associations between genotypes and RCC and ccRCC risk were assessed using additive and dominant models. Results of SNPs with a MAF < 0.25 were interpreted using a dominant model for power reasons. SNP allele frequencies in the subcohort were tested against departure from the Hardy-Weinberg Equilibrium using the Pearson χ^2^-test, as calculated with the Stata program ‘hwsnp’^45^. Gene-environment interactions were tested with the Wald χ^2^_-_test. Gene-environment analyses were adjusted for multiple comparisons with the adaptive Benjamini-Hochberg false discovery rate (FDR) procedure with a q-value threshold of 10%^30^. Sensitivity analyses were performed to explore the impact of using alternative categorizations for BMI (<20 kg/m2, 20–<25 kg/m2, 25–<30 kg/m2 and 30 + kg/m2), smoking status (never, ever), hypertension (no self-reported hypertension or no self-reported antihypertensive medication, hypertension with self-reported hypertensive medication) and alcohol consumption (0 g/d, 0.1–4 g/d, 5–14 g/d, 15–29 g/d and 30+ g/d) when assessing gene-environment interactions. Gene-gene interactions between *VHL* SNPs and the selected *HIF1A* SNP were tested using the Wald χ^2^_-_test. In a case-only analysis, the association between *VHL* SNPs and VHL tumor promoter methylation status (methylated, unmethylated) was assessed using multiple logistic regression for both RCC and ccRCC.

All analyses were performed using Stata Statistical Software: Release 15 (StataCorp., 2017, College Station, TX). The proportional hazards assumption was tested using scaled Schoenfeld residuals^46^. A violation of the assumption was apparent for age. Therefore, all models were adjusted for age as a time-dependent covariable. With the exception of FDR-corrected analyses, a p-value < 0.05 was considered statistically significant.

## Supplementary information

Supplementary Tables - 1, 2 and 3.

## Data Availability

The datasets generated during and/or analyzed during the current study are not publicly available because the participants did not provide written informed consent to the sharing of data.

## References

[1] Hsieh JJ (2017). Renal cell carcinoma. Nat Rev Dis Primers.

[2] Kaelin WG (2007). Von Hippel-Lindau disease. Annu Rev Pathol.

[3] Kim WY, Kaelin WG (2004). Role of VHL Gene Mutation in Human Cancer. Journal of Clinical Oncology.

[4] Nickerson ML (2008). Improved identification of von Hippel-Lindau gene alterations in clear cell renal tumors. Clin Cancer Res.

[5] van Houwelingen KP (2005). Prevalence of von Hippel-Lindau gene mutations in sporadic renal cell carcinoma: results from The Netherlands cohort study. BMC Cancer.

[6] Banks RE (2006). Genetic and epigenetic analysis of von Hippel-Lindau (VHL) gene alterations and relationship with clinical variables in sporadic renal cancer. Cancer Res.

[7] Gnarra JR (1994). Mutations of the VHL tumour suppressor gene in renal carcinoma. Nat Genet.

[8] Atlas Research N (2013). Comprehensive molecular characterization of clear cell renal cell carcinoma. Nature.

[9] Schodel J (2016). Hypoxia, Hypoxia-inducible Transcription Factors, and Renal Cancer. Eur Urol.

[10] Hunter DJ (2005). Gene-environment interactions in human diseases. Nat Rev Genet.

[11] Scelo G (2017). Genome-wide association study identifies multiple risk loci for renal cell carcinoma. Nat Commun.

[12] Henrion M (2013). Common variation at 2q22.3 (ZEB2) influences the risk of renal cancer. Hum Mol Genet.

[13] Purdue MP (2011). Genome-wide association study of renal cell carcinoma identifies two susceptibility loci on 2p21 and 11q13.3. Nat Genet.

[14] Wu X (2012). A genome-wide association study identifies a novel susceptibility locus for renal cell carcinoma on 12p11.23. Hum Mol Genet.

[15] Gudmundsson J (2013). A common variant at 8q24.21 is associated with renal cell cancer. Nat Commun.

[16] Purdue MP (2014). A genome-wide association study of renal cell carcinoma among African Americans. Cancer Epidemiol Biomarkers Prev.

[17] Henrion MY (2015). Common variation at 1q24.1 (ALDH9A1) is a potential risk factor for renal cancer. PLoS One.

[18] Laskar RS (2019). Sex specific associations in genome wide association analysis of renal cell carcinoma. Eur J Hum Genet.

[19] Wang WC, Tsou MH, Chen HJ, Hsu WF, Lai YC (2014). Two single nucleotide polymorphisms in the von Hippel-Lindau tumor suppressor gene in Taiwanese with renal cell carcinoma. BMC Res Notes.

[20] Lv C, Bai Z, Liu Z, Luo P, Zhang J (2015). Renal cell carcinoma risk is associated with the interactions of APOE, VHL and MTHFR gene polymorphisms. Int J Clin Exp Pathol.

[21] Qin C (2012). The polymorphisms in the VHL and HIF1A genes are associated with the prognosis but not the development of renal cell carcinoma. Ann Oncol.

[22] Qin C (2014). Variants in angiogenesis-related genes and the risk of clear cell renal cell carcinoma. Mutagenesis.

[23] Moore LE (2011). Von Hippel-Lindau (VHL) inactivation in sporadic clear cell renal cancer: associations with germline VHL polymorphisms and etiologic risk factors. PLoS Genet.

[24] Schouten LJ, Deckers IA, van den Brandt PA, Baldewijns MM, van Engeland M (2016). Alcohol and Dietary Folate Intake and Promoter CpG Island Methylation in Clear-Cell Renal Cell Cancer. Nutr Cancer.

[25] Smits KM (2008). Polymorphisms in genes related to activation or detoxification of carcinogens might interact with smoking to increase renal cancer risk: results from The Netherlands Cohort Study on diet and cancer. World J Urol.

[26] Antwi Samuel O., Eckel-Passow Jeanette E., Diehl Nancy D., Serie Daniel J., Custer Kaitlynn M., Wu Kevin J., Cheville John C., Thiel David D, Leibovich Bradley C., Parker Alexander S. (2017). Alcohol consumption, variability in alcohol dehydrogenase genes and risk of renal cell carcinoma. International Journal of Cancer.

[27] Melkonian SC (2016). Gene-environment interaction of genome-wide association study-identified susceptibility loci and meat-cooking mutagens in the etiology of renal cell carcinoma. Cancer.

[28] Karami S (2010). Vitamin d pathway genes, diet, and risk of renal cell carcinoma. Int J Endocrinol.

[29] Chow WH, Dong LM, Devesa SS (2010). Epidemiology and risk factors for kidney cancer. Nat Rev Urol.

[30] Benjamini Y, Hochberg Y (2000). On the Adaptive Control of the False Discovery Rate in Multiple Testing with Independent Statistics. Journal of Educational and Behavioral Statistics.

[31] Deckers IA (2015). Polymorphisms in genes of the renin-angiotensin-aldosterone system and renal cell cancer risk: interplay with hypertension and intakes of sodium, potassium and fluid. Int J Cancer.

[32] Schödel J (2012). Common genetic variants at the 11q13.3 renal cancer susceptibility locus influence binding of HIF to an enhancer of cyclin D1 expression. Nat Genet.

[33] van den Brandt PA (1990). A large-scale prospective cohort study on diet and cancer in The Netherlands. J Clin Epidemiol.

[34] Van den Brandt PA, Schouten LJ, Goldbohm RA, Dorant E, Hunen PM (1990). Development of a record linkage protocol for use in the Dutch Cancer Registry for Epidemiological Research. International Journal of Epidemiology.

[35] Goldbohm RA, Van den Brandt PA, Dorant E (1994). Estimation of the coverage of Dutch municipalities by cancer registries and PALGA based on hospital discharge data. Tijdschr Soc Gezondheidsz.

[36] Eble, J., Sauter, G., Epstein, J. & Sesterhenn, I. *World Health Organization Classification of Tumours. Pathology and Genetics*. Tumours of the Urinary System and Male Genital Organs. (IARC Press, 2004).

[37] Hogervorst JG (2014). DNA from nails for genetic analyses in large-scale epidemiologic studies. Cancer Epidemiol Biomarkers Prev.

[38] Cline RE, Laurent NM, Foran DR (2003). The fingernails of Mary Sullivan: developing reliable methods for selectively isolating endogenous and exogenous DNA from evidence. J Forensic Sci.

[39] Schouten LJ (2008). Alcohol consumption and mutations or promoter hypermethylation of the von Hippel-Lindau gene in renal cell carcinoma. Cancer Epidemiol Biomarkers Prev.

[40] Derks S (2004). Methylation-specific PCR unraveled. Cell Oncol.

[41] Herman JG, Graff JR, Myohanen S, Nelkin BD, Baylin SB (1996). Methylation-specific PCR: a novel PCR assay for methylation status of CpG islands. Proc Natl Acad Sci USA.

[42] van Engeland M (2002). K-ras mutations and RASSF1A promoter methylation in colorectal cancer. Oncogene.

[43] van den Brandt, P. A. & Goldbohm, R. A. *A prospective cohort study on diet and cancer in the Netherlands: design, conduct, analysis and first results after 3.3 years of follow-up*, Maastricht University (1993).

[44] Goldbohm RA (1994). Prospective study on alcohol consumption and the risk of cancer of the colon and rectum in the Netherlands. Cancer Causes Control.

[45] Cleves MA (2005). Exploratory analysis of single nucleotide polymorphism (SNP) for quantitative traits. Stata Journal.

[46] Schoenfeld D (1982). Partial Residuals for The Proportional Hazards Regression Model. Biometrika.

