# Supplementary material for: Germline polymorphisms in the Von Hippel-Lindau and Hypoxia-inducible factor 1-alpha genes, gene-environment and gene-gene interactions and renal cell cancer
Source: Sci Rep. 2020 Jan 10;10:137. doi: 10.1038/s41598-019-56980-0 (PMC6954183; doi:10.1038/s41598-019-56980-0)
Supplement: Supplementary file 1 — Supplementary Tables - 1, 2 and 3. [file 41598_2019_56980_MOESM1_ESM.docx]

**Supplemental files – Table 1, 2 & 3:**

**Germline polymorphisms in the *Von Hippel-Lindau* and *Hypoxia-inducible factor 1-alpha* genes, gene-environment and gene-gene interactions and renal cell cancer**

Jeroen A A van de Pol^a,*^, Piet A van den Brandt^a,b^, Manon van Engeland^c^, Roger W L Godschalk^d^, Frederik-Jan van Schooten^d^, Janneke G F Hogervorst^e^, Leo J Schouten^a^

^a^ Department of Epidemiology, GROW – School for Oncology and Developmental Biology, Maastricht University, Maastricht, the Netherlands

^b^ Department of Epidemiology, Care and Public Health Research Institute (CAPHRI), Maastricht University, Maastricht, the Netherlands

^c^ Department of Pathology, GROW – School for Oncology and Developmental Biology, Maastricht University Medical Centre, Maastricht, the Netherlands

^d^ Department of Pharmacology & Toxicology, NUTRIM School of Nutrition and Translational Research in Metabolism, Maastricht University, Maastricht, the Netherlands

^e^ Centre for Environmental Sciences, Hasselt University, Diepenbeek, Belgium

* Corresponding author: Jeroen A A van de Pol, Department of Epidemiology, Maastricht University, Peter Debyeplein 1, 6229 HA, Maastricht, the Netherlands. [jeroen.vandepol@maastrichtuniversity.nl](mailto:jeroen.vandepol@maastrichtuniversity.nl). Tel. +3143-3883423 Fax. +3143-3884128

ORCID: 0000-0002-2106-5634

Abbreviated title: *VHL* and *HIF1A* SNPs and RCC risk

**Supplementary Table 1 -** Descriptive characteristics of SNPs in subcohort members with ≥95% sample call rate; Netherlands Cohort Study on diet and cancer

|  |  |  |  |  | Genotype^a^ | | | | | |  |  |
| --- | --- | --- | --- | --- | --- | --- | --- | --- | --- | --- | --- | --- |
|  |  |  |  |  | 11 | | 12 | | 22 | | HWE^b^ |  |
| SNP | Gene | Chr. Location | Ref. Alleles | Minor Allele | n | % | n | % | n | % | p-value | MAF^c^ |
| rs1642739 | *VHL* | 3p25.3 | G/T | T | 2283 | 76.00 | 664 | 22.10 | 57 | 1.90 | 0.291 | 0.13 |
| rs779805 | *VHL* | 3p25.3 | A/G | G | 1414 | 47.07 | 1303 | 43.38 | 287 | 9.55 | 0.598 | 0.31 |
| rs265318 | *VHL* | 3p25.3 | A/C | C | 2401 | 79.93 | 569 | 18.94 | 34 | 1.13 | 0.965 | 0.11 |
| rs2301111 | *HIF1A* | 14q23.2 | C/G | G | 1895 | 63.08 | 988 | 32.89 | 121 | 4.03 | 0.581 | 0.20 |

^a^ 11: homozygous for major allele, 12: heterozygous for major and minor alleles, 22: homozygous for minor allele

^b^ Hardy-Weinberg Equilibrium as tested with Pearson χ^2^ test

^c^ Minor Allele Frequency

**Supplementary Table 2** - Sensitivity analyses for multivariable-adjusted gene-environment interactions for renal cell carcinoma (RCC) and clear-cell renal cell carcinoma; Netherlands Cohort Study on diet and cancer, 1986-2006

| Gene | |  | Wild genotype | | |  | Rare genotype | | |  |  |
| --- | --- | --- | --- | --- | --- | --- | --- | --- | --- | --- | --- |
|  | Environment |  | Subcohort  Person- years | No. cases | HR_adjusted_^a,b^ (CI95) |  | Subcohort  Person-years | No. cases | HR_adjusted_^a,b^ (CI95) | *P* for interaction^c^ | FDR *p*-threshold |
| **Renal cell carcinoma** | | | | | | | | | | |  |
| *VHL_*rs1642739 | |  | *GG* | | |  | *GT + TT* | | | |  |
|  | Hypertension | No | 33303 | 85 | 1 |  | 10653 | 10 | 1 |  |  |
|  | Incl. hypertensive meds | Yes | 5764 | 22 | 1.71 (1.26-2.33) |  | 1602 | 4 | 1.90 (1.09-3.29) | 0.855 | 0.081 |
|  | Smoking status^d^ | Never | 15367 | 72 | 1 |  | 4667 | 37 | 1 |  |  |
|  |  | Ever | 23700 | 227 | 1.41 (1.02-1.95) |  | 7588 | 70 | 1.19 (0.75-1.88) | 0.046 | 0.013 |
|  | BMI | <20 kg/m^2^ | 1210 | 4 | 0.61 (0.22-1.72) |  | 422 | 2 | 0.62 (0.14-2.68) |  |  |
|  |  | 20-<25 | 20729 | 138 | 1 |  | 5906 | 49 | 1 |  |  |
|  |  | 25-<30 | 14748 | 141 | 1.32 (1.02-1.71) |  | 5024 | 50 | 1.08 (0.68-1.73) |  |  |
|  |  | 30+ | 2380 | 16 | 1.02 (0.57-1.83) |  | 903 | 6 | 0.74 (0.47-1.18) | 0.858 | 0.088 |
|  | Alcohol intake | 0 g/d | 9220 | 59 | 1 |  | 2630 | 29 | 1 |  |  |
|  |  | 0.1-4 g/d | 11481 | 72 | 0.91 (0.63-1.31) |  | 3732 | 33 | 0.75 (0.43-1.32) |  |  |
|  |  | 5-14 g/d | 8695 | 60 | 0.79 (0.54-1.17) |  | 2646 | 24 | 0.68 (0.35-1.33) |  |  |
|  |  | 15-29 g/d | 6132 | 72 | 1.17 (0.79-1.74) |  | 2083 | 13 | 0.41 (0.18-0.92) |  |  |
|  |  | >=30 g/d | 3540 | 36 | 0.89 (0.55-1.44) |  | 1164 | 8 | 0.42 (0.16-1.08) | 0.019 | 0.006 |
| *VHL_*rs779805 | |  | *AA* | | |  | *AG + GG* | | | |  |
|  | Hypertension | No | 20435 | 127 | 1 |  | 23521 | 192 | 1 |  |  |
|  | Incl. hypertensive meds | Yes | 3473 | 30 | 1.62 (1.04-2.52) |  | 3892 | 57 | 1.79 (1.27-2.52) | 0.529 | 0.056 |
|  | Smoking status^d^ | Never | 9542 | 37 | 1 |  | 10492 | 72 | 1 |  |  |
|  |  | Ever | 14366 | 120 | 1.34 (0.86-2.08) |  | 16922 | 177 | 1.33 (0.95-1.86) | 0.137 | 0.031 |
|  | BMI | <20 kg/m^2^ | 820 | 3 | 0.87 (0.26-2.90) |  | 811 | 3 | 0.51 (0.16-1.66) |  |  |
|  |  | 20-<25 | 12555 | 71 | 1 |  | 14080 | 116 | 1 |  |  |
|  |  | 25-<30 | 8902 | 75 | 1.40 (0.98-2.00) |  | 10870 | 116 | 1.18 (0.89-1.57) |  |  |
|  |  | 30+ | 1631 | 8 | 1.07 (0.47-2.39) |  | 1652 | 14 | 0.91 (0.49-1.69) | 0.898 | 0.093 |
|  | Alcohol intake | 0 g/d | 5691 | 25 | 1 |  | 6159 | 63 | 1 |  |  |
|  |  | 0.1-4 g/d | 7250 | 39 | 1.08 (0.63-1.83) |  | 7963 | 66 | 0.78 (0.54-1.14) |  |  |
|  |  | 5-14 g/d | 5035 | 34 | 1.12 (0.63-1.97) |  | 6306 | 50 | 0.61 (0.40-0.93) |  |  |
|  |  | 15-29 g/d | 3796 | 39 | 1.40 (0.78-2.49) |  | 4419 | 46 | 0.71 (045-1.11) |  |  |
|  |  | >=30 g/d | 2137 | 20 | 1.09 (0.55-2.17) |  | 2567 | 24 | 0.59 (0.34-1.03) | 0.096 | 0.025 |
| *VHL_*rs265318 | |  | *AA* | | |  | *AC + CC* | | | |  |
|  | Hypertension | No | 35163 | 251 | 1 |  | 8793 | 68 | 1 |  |  |
|  | Incl. hypertensive meds | Yes | 5903 | 70 | 1.77 (1.31-2.37) |  | 1463 | 17 | 1.69 (0.90-3.16) | 0.705 | 0.069 |
|  | Smoking status^d^ | Never | 16103 | 85 | 1 |  | 3932 | 24 | 1 |  |  |
|  |  | Ever | 24964 | 236 | 1.30 (0.96-1.76) |  | 6324 | 61 | 1.41 (0.82-2.42) | 0.815 | 0.075 |
|  | BMI | <20 kg/m^2^ | 1294 | 5 | 0.71 (0.28-1.79) |  | 337 | 1 | 0.40 (0.53-3.00) |  |  |
|  |  | 20-<25 | 21654 | 143 | 1 |  | 4981 | 44 | 1 |  |  |
|  |  | 25-<30 | 15468 | 155 | 1.40 (1.09-1.79) |  | 4304 | 36 | 0.84 (0.49-1.43) |  |  |
|  |  | 30+ | 2650 | 18 | 1.03 (0.60-1.78) |  | 634 | 4 | 0.78 (0.25-2.40) | 0.307 | 0.044 |
|  | Alcohol intake | 0 g/d | 9649 | 67 | 1 |  | 2201 | 21 | 1 |  |  |
|  |  | 0.1-4 g/d | 12140 | 80 | 0.89 (0.63-1.26) |  | 3073 | 25 | 0.78 (0.41-1.48) |  |  |
|  |  | 5-14 g/d | 9071 | 66 | 0.81 (0.56-1.17) |  | 2270 | 18 | 0.64 (0.31-1.35) |  |  |
|  |  | 15-29 g/d | 6514 | 72 | 1.06 (0.72-1.55) |  | 1701 | 13 | 0.54 (0.23-1.28) |  |  |
|  |  | >=30 g/d | 3693 | 36 | 0.83 (0.52-1.34) |  | 1011 | 8 | 0.50 (0.18-1.37) | 0.503 | 0.050 |
| *HIF1A*_rs2301111 | |  | *CC* | | |  | *CG + GG* | | | |  |
|  | Hypertension | No | 27916 | 202 | 1 |  | 16040 | 117 | 1 |  |  |
|  | Incl. hypertensive meds | Yes | 4636 | 55 | 1.71 (1.23-2.38) |  | 2729 | 32 | 1.79 (1.13-2.83) | 0.901 | 0.100 |
|  | Smoking status^d^ | Never | 12704 | 77 | 1 |  | 7330 | 32 | 1 |  |  |
|  |  | Ever | 19849 | 180 | 1.11 (0.80-1.53) |  | 11439 | 117 | 1.88 (1.19-2.96) | 0.061 | 0.019 |
|  | BMI | <20 kg/m^2^ | 1085 | 4 | 0.66 (0.24-1.85) |  | 547 | 2 | 0.59 (0.14-2.51) |  |  |
|  |  | 20-<25 | 16712 | 115 | 1 |  | 9923 | 72 | 1 |  |  |
|  |  | 25-<30 | 12538 | 126 | 1.35 (1.02-1.79) |  | 7234 | 65 | 1.17 (0.81-1.70) |  |  |
|  |  | 30+ | 218 | 12 | 0.80 (0.41-1.53) |  | 1065 | 10 | 1.36 (0.64-2.88) | 0.569 | 0.063 |
|  | Alcohol intake | 0 g/d | 7176 | 54 | 1 |  | 4674 | 34 | 1 |  |  |
|  |  | 0.1-4 g/d | 9527 | 72 | 0.92 (0.63-1.36) |  | 5685 | 33 | 0.75 (0.45-1.26) |  |  |
|  |  | 5-14 g/d | 7321 | 58 | 0.79 (0.52-1.20) |  | 4020 | 26 | 0.72 (0.41-1.25) |  |  |
|  | Hypertension | 15-29 g/d | 5264 | 46 | 0.77 (0.49-1.23) |  | 2951 | 39 | 1.16 (0.67-2.01) |  |  |
|  |  | >=30 g/d | 3265 | 27 | 0.65 (0.38-1.11) |  | 1438 | 17 | 0.97 (0.48-1.95) | 0.224 | 0.038 |
| **Clear cell Renal Cell Carcinoma** | | | | | | | | | | |  |
| *VHL_*rs1642739 | |  | *GG* | | |  | *GT + TT* | | | |  |
|  | Hypertension | No | 33303 | 148 | 1 |  | 10653 | 59 | 1 |  |  |
|  | Incl. hypertensive meds | Yes | 5764 | 42 | 1.71 (1.18-2.48) |  | 1602 | 14 | 1.88 (0.96-3.69) | 0.930 | 0.088 |
|  | Smoking status^d^ | Never | 15367 | 47 | 1 |  | 4667 | 23 | 1 |  |  |
|  |  | Ever | 23700 | 143 | 1.45 (0.98-2.15) |  | 7588 | 50 | 1.35 (0.79-2.32) | 0.287 | 0.031 |
|  | BMI | <20 kg/m^2^ | 1210 | 3 | 0.76 (0.23-2.49) |  | 422 | 1 | 0.46 (0.06-3.47) |  |  |
|  |  | 20-<25 | 20729 | 83 | 1 |  | 5906 | 32 | 1 |  |  |
|  |  | 25-<30 | 14748 | 93 | 1.45 (1.06-1.98) |  | 5024 | 38 | 1.34 (0.80-2.24) |  |  |
|  |  | 30+ | 2380 | 11 | 1.14 (0.56-2.29) |  | 903 | 2 | 0.43 (0.10-1.89) | 0.720 | 0.069 |
|  | Alcohol intake | 0 g/d | 9220 | 40 | 1 |  | 2630 | 20 | 1 |  |  |
|  |  | 0.1-4 g/d | 11481 | 40 | 0.74 (0.47-1.17) |  | 3732 | 26 | 0.81 (0.43-1.55) |  |  |
|  |  | 5-14 g/d | 8695 | 40 | 0.80 (0.51-1.26) |  | 2646 | 14 | 0.51 (0.22-1.18) |  |  |
|  |  | 15-29 g/d | 6132 | 51 | 1.29 (0.81-2.05) |  | 2083 | 6 | 0.24 (0.08-0.67) |  |  |
|  |  | >=30 g/d | 3540 | 19 | 0.74 (0.40-1.37) |  | 1164 | 7 | 0.48 (0.17-1.38) | 0.009 | 0.006 |
| *VHL_*rs779805 | |  | *AA* | | |  | *AG + GG* | | | |  |
|  | Hypertension | No | 20435 | 79 | 1 |  | 23521 | 128 | 1 |  |  |
|  | Incl. hypertensive meds | Yes | 3473 | 19 | 1.76 (1.17-2.64) |  | 3892 | 37 | 1.41 (1.05-1.89) | 0.741 | 0.075 |
|  | Smoking status^d^ | Never | 9542 | 21 | 1 |  | 10492 | 49 | 1 |  |  |
|  |  | Ever | 14366 | 77 | 1.65 (0.93-2.90) |  | 16922 | 116 | 1.31 (0.88-1.93) | 0.084 | 0.019 |
|  | BMI | <20 kg/m^2^ | 820 | 3 | 1.50 (0.44-5.12) |  | 811 | 1 | 0.26 (0.04-1.93) |  |  |
|  |  | 20-<25 | 12555 | 41 | 1 |  | 14080 | 74 | 1 |  |  |
|  |  | 25-<30 | 8902 | 48 | 1.56 (1.00-2.42) |  | 10870 | 83 | 1.35 (0.97-1.89) |  |  |
|  |  | 30+ | 1631 | 6 | 1.33 (0.51-3.49) |  | 1652 | 7 | 0.75 (0.32-1.74) | 0.518 | 0.063 |
|  | Alcohol intake | 0 g/d | 5691 | 15 | 1 |  | 6159 | 45 | 1 |  |  |
|  |  | 0.1-4 g/d | 7250 | 22 | 1.01 (0.51-1.98) |  | 7963 | 44 | 0.71 (0.46-1.10) |  |  |
|  |  | 5-14 g/d | 5035 | 25 | 1.36 (0.69-2.68) |  | 6306 | 29 | 0.48 (0.29-0.81) |  |  |
|  |  | 15-29 g/d | 3796 | 24 | 1.45 (0.70-2.68) |  | 4419 | 33 | 0.71 (0.43-1.20) |  |  |
|  |  | >=30 g/d | 2137 | 12 | 1.15 (0.49-2.73) |  | 2567 | 14 | 0.49 (0.25-0.97) | 0.049 | 0.013 |
| *VHL_*rs265318 | |  | *AA* | | |  | *AC + CC* | | | |  |
|  | Hypertension | No | 35163 | 162 | 1 |  | 8793 | 45 | 1 |  |  |
|  | Incl. hypertensive meds | Yes | 5903 | 44 | 1.71 (1.19-2.45) |  | 1463 | 12 | 1.86 (0.90-3.85) | 0.955 | 0.094 |
|  | Smoking status^d^ | Never | 16103 | 54 | 1 |  | 3932 | 16 | 1 |  |  |
|  |  | Ever | 24964 | 152 | 1.36 (0.94-1.97) |  | 6324 | 41 | 1.53 (0.81-2.88) | 0.847 | 0.081 |
|  | BMI | <20 kg/m^2^ | 1294 | 4 | 0.94 (0.33-2.63) |  | 337 | - | - |  |  |
|  |  | 20-<25 | 21654 | 88 | 1 |  | 4981 | 27 | 1 |  |  |
|  |  | 25-<30 | 15468 | 103 | 1.51 (1.12-2.04) |  | 4304 | 28 | 1.17 (0.66-2.08) |  |  |
|  |  | 30+ | 2650 | 11 | 1.01 (0.51-2.02) |  | 634 | 2 | 0.69 (0.15-3.19) | * | 0.100 |
|  | Alcohol intake | 0 g/d | 9649 | 44 | 1 |  | 2201 | 16 | 1 |  |  |
|  |  | 0.1-4 g/d | 12140 | 47 | 0.80 (0.52-1.22) |  | 3073 | 19 | 0.75 (0.36-1.55) |  |  |
|  |  | 5-14 g/d | 9071 | 43 | 0.80 (0.52-1.25) |  | 2270 | 11 | 0.48 (0.20-1.17) |  |  |
|  |  | 15-29 g/d | 6514 | 51 | 1.16 (0.74-1.83) |  | 1701 | 6 | 0.31 (0.10-0.91) |  |  |
|  |  | >=30 g/d | 3693 | 21 | 0.76 (0.42-1.37) |  | 1011 | 5 | 0.42 (0.13-1.42) | 0.120 | 0.025 |
| *HIF1A*_rs2301111 | |  | *CC* | | |  | *CG + GG* | | | |  |
|  | Hypertension | No | 27916 | 137 | 1 |  | 16040 | 70 | 1 |  |  |
|  | Incl. hypertensive meds | Yes | 4636 | 33 | 1.53 (1.02-2.29) |  | 2729 | 23 | 2.12 (1.23-3.65) | 0.290 | 0.038 |
|  | Smoking status^d^ | Never | 12704 | 48 | 1 |  | 7330 | 22 | 1 |  |  |
|  |  | Ever | 19849 | 122 | 1.26 (0.86-1.85) |  | 11439 | 71 | 1.74 (1.00-3.05) | 0.366 | 0.040 |
|  | BMI | <20 kg/m^2^ | 1085 | 3 | 0.76 (0.24-2.48) |  | 547 | 1 | 0.51 (0.07-3.92) |  |  |
|  |  | 20-<25 | 16712 | 74 | 1 |  | 9923 | 41 | 1 |  |  |
|  |  | 25-<30 | 12538 | 87 | 1.47 (1.05-2.05) |  | 7234 | 44 | 1.37 (0.87-2.14) |  |  |
|  |  | 30+ | 218 | 6 | 0.61 (0.25-1.48) |  | 1065 | 7 | 1.64 (0.67-4.05) | 0.411 | 0.056 |
|  | Alcohol intake | 0 g/d | 7176 | 36 | 1 |  | 4674 | 24 | 1 |  |  |
|  |  | 0.1-4 g/d | 9527 | 45 | 0.86 (0.54-1.36) |  | 5685 | 21 | 0.67 (0.36-1.23) |  |  |
|  |  | 5-14 g/d | 7321 | 40 | 0.80 (0.49-1.31) |  | 4020 | 14 | 0.53 (0.26-1.08) |  |  |
|  |  | 15-29 g/d | 5264 | 31 | 0.78 (0.45-1.35) |  | 2951 | 26 | 1.13 (0.58-2.18) |  |  |
|  |  | >=30 g/d | 3265 | 18 | 0.66 (0.35-1.25) |  | 1438 | 8 | 0.70 (0.28-1.77) | 0.386 | 0.050 |

^a^ Models include a time-varying covariable for age due to a probable violation of the proportional hazards assumption.

^b^ Models adjusted for age (y, continuous), sex (man/woman), hypertension (yes/no), smoking status (never, former, current), smoking duration (y, centered), smoking intensity (cig/d, centered), BMI (kg/m^2^, continuous) and alcohol consumption (g ethanol/d, continuous) when applicable

^c^ No interaction was significant after false discovery rate correction

^d^ Additionally adjusted for smoking duration (centered, years) and smoking intensity (centered, cig/d)

**Supplementary Table 3:** Case-only analysis on the association between *VHL* promoter methylation status and *VHL* SNP status, the Netherlands Cohort Study on Diet and Cancer, 1986-2006.

|  | ***VHL* promoter methylation status** | | **Age and sex-adjusted** | **Multivariable-adjusted** |
| --- | --- | --- | --- | --- |
|  | **Methylated** | **Unmethylated** | **OR^a^ (95% CI)** | **OR^b^ (95% CI)** |
| **Clear-cell renal cell carcinoma** | | | | |
| VHL - rs1642739 |  |  |  |  |
| GG | 16 | 169 | 1 | 1 |
| GT+TT | 3 | 65 | 0.48 (0.14-1.73) | 0.45 (0.12-1.69) |
| VHL - rs779805 |  |  |  |  |
| AA | 7 | 88 | 1 | 1 |
| AG+GG | 12 | 146 | 1.02 (0.38-2.70) | 0.99 (0.37-2.69) |
| VHL - rs265318 |  |  |  |  |
| AA | 17 | 183 | 1 | 1 |
| AC+CC | 2 | 51 | 0.42 (0.09-1.87) | 0.38 (0.07-2.00) |
| Total number of SNPs present | | | | |
| None | 6 | 85 | 1 | 1 |
| 1 or more | 13 | 150 | 1.20 (0.43-3.36) | 1.13 (0.39-3.29) |

^a^ Models adjusted for age (y, continuous) and sex (man/woman)

^b^ Models adjusted for age (y, continuous), sex (man/woman), hypertension (yes/no), smoking status (never, former, current), smoking duration (y, centered), smoking intensity (cig/d, centered), BMI (kg/m^2^, continuous) and alcohol intake (g ethanol/d, continuous)
